# Autoinducer-2 Quorum Sensing Contributes to Regulation of Microcin PDI in *Escherichia coli*

**DOI:** 10.3389/fmicb.2017.02570

**Published:** 2017-12-22

**Authors:** Shao-Yeh Lu, Zhe Zhao, Johannetsy J. Avillan, Jinxin Liu, Douglas R. Call

**Affiliations:** ^1^Paul G. Allen School for Global Animal Health, Washington State University, Pullman, WA, United States; ^2^Institute of Marine Biology, College of Oceanography, Hohai University, Nanjing, China; ^3^Department of Food Science and Technology, University of California, Davis, Davis, CA, United States

**Keywords:** autoinducer-2, bacteriocin, microcin, mccPDI, *luxS*, *lsr*, quorum sensing

## Abstract

The *Escherichia coli* quorum sensing (QS) signal molecule, autoinducer-2 (AI-2), reaches its maximum concentration during mid-to-late growth phase after which it quickly degrades during stationary phase. This pattern of AI-2 concentration coincides with the up- then down-regulation of a recently described microcin PDI (mccPDI) effector protein (McpM). To determine if there is a functional relationship between these systems, a prototypical mccPDI-expressing strain of *E. coli* 25 was used to generate Δ*luxS*, Δ*lsrACDBFG* (Δ*lsr*), and Δ*lsrR* mutant strains that are deficient in AI-2 production, transportation, and AI-2 transport regulation, respectively. Trans-complementation, RT-qPCR, and western blot assays were used to detect changes of microcin expression and synthesis under co-culture and monoculture conditions. Compared to the wild-type strain, the AI-2-deficient strain (Δ*luxS*) and -uptake negative strain (Δ*lsr*) were >1,000-fold less inhibitory to susceptible bacteria (*P* < 0.05). With *in trans* complementation of *luxS*, the AI-2 deficient mutant reduced the susceptible *E. coli* population by 4-log, which was within 1-log of the wild-type phenotype. RT-qPCR and western blot results for the AI-2 deficient *E. coli* 25 showed a 5-fold reduction in *mcpM* transcription with an average 2-h delay in McpM synthesis. Furthermore, overexpression of sRNA *micC* and *micF* (both involved in porin protein regulation) was correlated with *mcpM* regulation, consistent with a possible link between QS and *mcpM* regulation. This is the direct first evidence that microcin regulation can be linked to quorum sensing in a Gram-negative bacterium.

## Introduction

Bacteria can regulate specific cellular functions through quorum sensing (QS), which is a density-dependent, cell-to-cell communication system (Papenfort and Bassler, 2016). In response to changes in cell density, QS allows bacteria to alter behavior and regulate global gene expression collectively through the accumulation of threshold concentrations of small, diffusible autoinducer (AI) signal molecules (Papenfort and Bassler, 2016). Both Gram-negative and -positive bacterial species can produce QS signaling molecules such as autoinducer-2 (AI-2), which in some bacterial species can affect inter- and intra-specific behavior (Sun et al., 2004; Federle, 2009; Xue et al., 2009). For example, AI-2 contributes to gene regulation for *E. coli* O157:H7 including regulation of virulence gene expression (Sperandio et al., 2002), type III secretion (Sperandio et al., 1999), flagellar synthesis, motility, and chemotaxis (Sperandio et al., 2001). Moreover, at high cell density, *E. coli* AI-2 can bind to cellular receptors that subsequently regulate protein production and biofilm formation (DeLisa et al., 2001). During the mid-to-late exponential growth phase, AI-2 reaches its maximum concentration followed by degradation during the stationary phase (Surette and Bassler, 1998; Ren et al., 2004). This temporal pattern of AI-2 concentration coincides with the up- and down-regulation of the recently described microcin PDI (mccPDI) in *E. coli* (Eberhart et al., 2012).

MccPDI was first described from a cattle *E. coli* isolate 25 (*E. coli* 25) and it inhibits a diversity of *E. coli* strains including enterohemorrhagic *E. coli* (EHEC) serotypes O157:H7 and O26 (Eberhart et al., 2012, 2014; Zhao et al., 2015). The inhibitory phenotype was characterized as “proximity-dependent inhibition” (PDI) due to the apparent need for the producing strain to be in close proximity to inhibit susceptible cells (Sawant et al., 2011; Eberhart et al., 2012). Zhao et al. (2017) previously showed that in the presence of low osmolarity conditions, synthesis of the mccPDI effector protein (McpM) is upregulated via a two-component regulatory system, EnvZ/OmpR (Zhao et al., 2017). Maximal inhibition from PDI occurs during the mid-to-late exponential growth phase, but declines rapidly during stationary phase despite continuing low-osmolarity conditions in the growth media. The fact that temporal expression of microcin PDI coincides with the maximum concentration of AI-2 at mid-to-late-exponential growth phase and degradation of AI-2 in stationary phase suggests the possibility that AI-2 QS plays a role in the PDI regulation. Consequently, we hypothesized that bacteria's ability to detect cell-to-cell density through AI-2 QS contributes to regulation of the mccPDI phenotype. Through a series of gene knockout and complementation experiments, we found that a PDI-positive strain that was deficient in the QS system was also defective for inhibition of susceptible bacteria (*E. coli* K-12 BW25113), *mcpM* transcription, and delayed McpM synthesis in comparison to the wild-type strain. These findings highlight the complexity of microcin PDI regulation and contribute to the understanding of the regulatory mechanisms of Class IIa microcins in *E. coli*.

## Materials and methods

### Bacterial strains and culture conditions

Unless otherwise stated, the *E. coli* strains used in this study (Table 1) were grown in LB -Lennox (LB broth) medium (Difco) or in M9 minimal defined medium (Na_2_HPO_4_ 6 g/L, KH_2_PO_4_ 3 g/L, NaCl 0.5 g/L, NH_4_Cl 1 g/L, MgSO_4_ 1 mM, CaCl_2_ 0.1 mM, and 0.2% glucose) supplemented with thiamine (1 mg/L) and leucine (100 μg/mL; Eberhart et al., 2012; Zhao et al., 2015, 2017) at 37°C with shaking at 200 rpm. Antibiotics were added to media as needed (ampicillin, Amp, 100 μg/mL; tetracycline, Tet, 50 μg/mL; kanamycin, Kan, 50 μg/mL; nalidixic acid, Nal, 30 μg/mL; chloramphenicol, Cm, 32 μg/mL). *Vibrio harveyi* MM32 (ATCC BAA-1121) (Table 1) was grown in marine broth 2216 (Difco) or autoinducer bioassay (AB) medium [NaCl 17.5 g/L, MgSO_4_ 12.3 g/L, casamino acids (vitamin-free) 2.0 g/L, KH_2_PO_4_ (pH 7.0) 1 M, L-arginine 0.1 M, and glycerol 10 mL/L; (ATCC)] at 30°C with shaking at 200 rpm and antibiotics were added as needed (Amp, 50 μg/mL; Kan, 25 μg/mL; Cm, 15 μg/mL).

**Table 1:** Strains and plasmids used in this study.

**Strains/Plasmid name**	**Relevant genotype/phenotype^a^**	**References**
***Escherichia coli*** **STRAINS**		
**25**	Wild-type; SSuT^r^ PDI^+^	Sawant et al., 2011
25 Δ*luxS*	SSuT^r^ PDI^+^, *luxS* knockout	This study
25 Δ*lsr*	SSuT^r^ PDI^+^, *lsrACDBFG* knockout	This study
25 Δ*lsrK*	SSuT^r^ PDI^+^, *lsrK* knockout	This study
25 Δ*lsrR*	SSuT^r^ PDI^+^, *lsrR* knockout	This study
25 Δ*mcpM*	SSuT^r^ PDI^+^, *mcpM* knockout	Zhao et al., 2017
25 Δ*mcpM*/pCR2.1::P_mic−10/−210_*mcpM*	SSuT^r^ PDI^+^, *mcpM* knockout complemented with *mcpM* driven by endogenous promoter	Zhao et al., 2017
25 Δ*luxS*/pCR2.1::P_mic−10/−210_*mcpM*	SSuT^r^ PDI^+^, Cm^r^, *luxS* knockout complemented with *mcpM* driven by endogenous promoter	This study
25 Δ*luxS*/pBAD18-Cm::*luxS*	SSuT^r^ PDI^+^, Cm^r^, *luxS* knockout complemented with *luxS* driven by *araC* promoter	This study
25 Δ*luxS*/pBAD18-Cm	SSuT^r^ PDI^+^, Cm^r^, *luxS* knockout complemented with empty pBAD18-Cm vector	This study
25 Δ*ompR*	SSuT^r^ PDI^+^, *ompR* knockout	Zhao et al., 2015
25/pGEM-2	SSuT^r^ PDI^+^, Amp^r^, complemented with empty pGEM-2 vector	This study
25/pGEM-2-*micF*	SSuT^r^ PDI^+^, Amp^r^, complemented with *micF* driven by T7 promoter	This study
25/pGEM-2-*micC*	SSuT^r^ PDI^+^, Amp^r^, complemented with *micC* driven by T7 promoter	This study
**BW25113**	Nal^r^, Keio collection wild-type K-12 strain	Baba et al., 2006
BW25113 Δ*luxS*	Kan^r^, Keio collection, *luxS* knockout	Baba et al., 2006
**S17-1**Δ ***pir***	*thi pro hsdR hsdM*^+^*recA* RP4-2-Tc::Mu-Km::Tn*7 Δ p*ir lysogen	Simon et al., 1983
S17/pDM4-Δ*mcpM*	S17 strain carrying the plasmid pDM4-Δ*mcpM*	Zhao et al., 2017
***Vibrio harveyi*** **STRAINS**		
MM32 (ATCC BAA-1121)	BB120 *luxN*::Cm, *luxS*::Tn5Kan; AI-1^+^, AI-2^−^	ATCC, Miller et al., 2004
**PLASMIDS**		
**pCR2.1-TOPO vector (pCR2.1)**	Amp^r^, cloning vector	Invitrogen
pCR2.1::P_mic−10/−210_*mcpM*	Amp^r^, pCR2.1 containing the *mcpM* gene with 6x His.tag at the C-terminus under the endogenous promoter control	Zhao et al., 2017
**pBAD18-Cm vector (pBAD18-Cm)**	Cm^r^, expression vector under the *araC* promoter control	Guzman et al., 1995
pBAD18-Cm::*luxS*	Cm^r^, pBAD18-Cm containing the *luxS* gene with 6x His.tag at the C-terminus under the *araC* promoter control	This study
**pDM4 vector**	Cm^r^, Suicide vector with an R6K origin (*pir*-requiring) and *sacBR* of *Bacillus subtilis*	Milton et al., 1996
pDM4-Δ*mcpM*	Cm^r^, pDM4 containing the flanking region sequences of *mcpM*	Zhao et al., 2017
**pKD46**	Amp^r^	Datsenko and Wanner, 2000
**pKD4**	Kan^r^, containing Kan^r^ cassette for PCR amplification	Datsenko and Wanner, 2000
**pGEM-2**	Amp^r^, pGEM-2 cloning vector	Promega
pGEM-2-*micC*	Amp^r^, pGEM-2 containing the *micC* gene insert	Chen et al., 2004
pGEM-2-*micF*	Amp^r^, pGEM-2 containing the *micF* gene insert	Chen et al., 2004

a *Amp^r^, Ampicillin resistant; Cm^r^, chloramphenicol resistant; Kan^r^, Kanamycin resistant; Nal^r^, nalidixic acid resistant; SSuT^r^, streptomycin, sulfadiazine, and tetracycline resistant*.

### Plasmid extraction and vector construction

All plasmids were extracted from *E. coli* by using a QIAprep Spin Miniprep kit (Qiagen). *E. coli* genomic DNA was extracted with the DNeasy Blood & Tissue kit (Qiagen). Platinum PCR Super Mix (Invitrogen) was used for preparative PCR when working with plasmid pBAD18-Cm, pDM4, pGEM-2, and pKD4. Complementation were performed using primers incorporating restriction sites (Supplemental Table 1) for PCR amplification, restriction digest (New England Biolabs Inc.), and ligation (T4 ligase, New England Biolabs Inc.) following standard cloning techniques. All conventional PCR for verification of constructs and gene detection used DreamTag Green PCR Master Mix (Thermo Scientific) and PCR products were confirmed by sequencing (Eurofins Genomics).

### Mutant construction

Gene-specific PCR-mediated gene deletion followed the methods of Datsenko and Wanner (Datsenko and Wanner, 2000). Briefly, primers (Supplemental Table 1) were designed to incorporate a 36- to 50-nucleotide segment that was complementary to the DNA sequence flanking the gene of interest. Primers were used to generate a PCR product that joined these flanking sequences to a Kan-resistance gene (*kan*^r^) that originated from pKD4 (Table 1). PCR products were column purified by using a QIAquick PCR purification kit (Qiagen). Restriction enzyme (DpnI; New England Biolabs Inc.) was used to digest pKD4 plasmid for 4 h at 37°C before column purification was repeated. Processed PCR products (150 ng) were then suspended in 5 μL of 10 mM Tris (pH 8.0) and were electroporated into *E. coli* 25 with a Gene Pulser Xcell (Bio-Rad) as described previously (Zhao et al., 2015). Briefly, *E. coli* 25 carrying the λ Red plasmid pKD46 (Amp^r^) was prepared for electroportation (1.8 kV, 25 μF, 200 Ω, 1 mm gap cuvette) by first growing culture to an optical density (OD_600nm_) of ~0.6 in SOB medium (Fisher Scientific) (Table 1) with 1 mM _L_-arabinose (30°C). Cells were then washed twice in ice-cold water and once in 10% glycerol. Cells were subsequently resuspended in 10% glycerol (50 μL) for electroporation. Immediately after electroporation, cells were resuspended in SOC recovery medium (Fisher Scientific) for 2 h at 30°C (200 rpm) before plating on Kan-containing LB agar and incubating overnight at 30°C. PCR was used to verify gene deletion of *lsr, lsrK*, and *lsrR* (Table S1). All mutants were generated utilizing this method with the exception of *E. coli 25* Δ*luxS*, for which a splice-overlap-extension method was used (Heckman and Pease, 2007). Briefly, two 400- to 600-bp PCR fragments from sequences flanking *luxS* were joined and then cloned into a suicide plasmid (pDM4; Cm^r^; Table 1) by using standard cloning procedures (Milton et al., 1996). Constructs were confirmed by DNA sequencing (Eurofins Genomics) prior to electroporating into electrocompetent *E. coli* S17-1 λpir (Table 1). Conjugation was performed with *E. coli* 25 to generate a mutant that was selected on LB agar plates (Tet and Cm antibiotics) followed by a 10% sucrose selection (Zhao et al., 2015). PCR was used to confirm the deletion of *luxS*.

### RNA extraction and quantitative RT-PCR (RT-PCR)

Cultures (5 mL) were grown overnight in M9 medium and total RNA was extracted from an aliquot (1.5 mL) with the RiboPure^TM^-Bacteria kit (Ambion) per manufacturer's instruction with an additional DNase treatment with a RQ-1 RNase-Free DNase (Promega). RNA was quantified by using a NanoDrop^TM^ 2000 spectrophotometer (ThermoFisher Scientific). Complementary DNA was generated from DNase-treated total RNA (500 ng) with iScript Reverse Transcription Supermix (Bio-Rad) per manufacturer's instruction. Quantitative RT-PCR was completed in triplicate using the SsoAdvanced SYBR Green Supermix (Bio-Rad) per manufacturer's instruction with indicated primers (Supplemental Table 1). A CFX98 Real-Time System (Bio-Rad) was used to perform the thermal cycling parameters: one cycle at 95°C for 30 s; 39 cycles of 95°C for 5 s, 55°C for 15 s with plate read and 72°C for 30 s; 65°C for 5 s and plate read every 0.5°C/cycle to 95°C. The relative gene expression level was calculated with wild-type *E. coli* 25 serving as the control for calculations using the ΔΔCt method (Livak and Schmittgen, 2001). To detect potential DNA contamination, before the reverse transcription reaction an aliquot of each RNA extraction was subjected to conventional qPCR with *rpoD* primers (Cq-values >37, signified low level of DNA contamination).

### Co-culture competition assays

Co-culture competition assays were performed with a modified competition assay protocol (Chen et al., 2003; Zhao et al., 2015). Briefly, strains to be competed were grown individually in LB broth overnight. The next day the individual overnight cultures were combined (1:1) and inoculated into fresh M9 medium at a ratio of 1:100 for competition for 4, 8, 12, and 24 h at 37°C with aeration. Individual strains were also inoculated (monoculture) under the same conditions as controls. When appropriate, antibiotics and/or 0.2% (w/v) _L_-arabinose was added to pBAD18-Cm constructs or 0.5 mM Isopropyl β-D-1-thiogalactopyranoside (IPTG) to pGEM-2 constructs unless otherwise noted. Colony forming unit (CFU) were quantified by using serial dilution and a 6X6 drop-plate technique (Chen et al., 2003).

### Autoinducer bioassay

Measurement of AI-2 production by *E. coli* 25 and complemented *luxS* strains was done by using an autoinducer bioassay (AB) as previously described (Surette and Bassler, 1998, 1999). Strains of interest were grown overnight at 30°C with aeration in LB medium supplemented with 0.5% glucose. *V. harveyi* MM32 was grown overnight at 30°C with aeration in AB medium supplemented with 0.5% glucose. On the following day, bacterial cultures were inoculated (1:100) into fresh media (as described, respectively) and were grown for 8 h at 30°C with aeration. *E. coli* 25 Δ*luxS*/pBAD18-Cm and *E. coli* 25 Δ*luxS*/pBAD18-Cm::*luxS* were grown in LB without glucose to avoid *araC* inhibition during _L_-arabinose induction of pBAD18-Cm::*luxS* (Simcikova et al., 2014). Samples were centrifuged at 18,000 × g for 10 min and filtered (0.22 μm) to obtain cell-free supernatants that were stored at −20°C. The autoinducer bioassay (AB) medium (Bassler et al., 1993) was used to grow reporter strain *V. harveyi* MM32 (autoinducer 1^−^, autoinducer 2^−^; Bassler et al., 1993). Previously prepared cell-free supernatants were tested for the presence of AI-2 by adding to *V. harveyi* culture followed by detection of luminesce. Briefly, reporter strain *V. harveyi* MM32 was grown overnight in AB medium (30°C for 16 h) and was then diluted (1:5,000) in fresh AB medium. An aliquot (90 μL) was added to each well of a 96-well plate with 10 μL supernatant sample (from above). A positive-control well contained cell-free supernatant from *E. coli* 25 wild-type, while a negative-control well contained *V. harveyi* MM32 with no supernatant added. Plates were sealed with breathable sealing film (Axygen) and luminescence was measured every hour using an Infiniti M1000 PRO microplate reader (Tecan Systems). Each assay was repeated for three independent replicates.

### Protein analysis

Isolated colonies were inoculated into 5-mL LB media with appropriate antibiotic and grown as described. Overnight culture was diluted (1:100) into fresh M9 media (10 mL) and grown overnight at 37°C with 200 rpm shaking until OD600 ~ 0.6 at which point 0.02% (w/v) _L_-arabinose was added for 24 h at room temperature with shaking at 200 rpm. Total proteins were collected by centrifugation at 18,000 × g at 4°C for 5 min. Cell pellets were resuspended in 1x laemmli sample buffer (Bio-Rad) and boiled for 10 min. Any kD Tris-glycine precast gels (Bio-Rad) were used for SDS-PAGE protein separation. A Trans-Blot turbo transfer starter system (Bio-Rad) was used to transfer proteins onto a low-fluorescence polyvinylidene fluoride membrane (Bio-Rad) and Ponceau S stain was used to verify protein transfer prior to addition of antibodies for specific protein detection. Primary antibody anti-His-tag (1:1,000; Thermo Scientific) was used with secondary goat anti-mouse antibody (1:5,000; DyLight 650, conjugate). A ChemiDoc MP Imaging System (Bio-Rad) was used to detect fluorescent signal and band intensity was quantified with ImageJ software (Schneider et al., 2012). A ratio of McpM value to DnaK value served to normalize and quantify and are represented by arbitrary unit (AU).

### Statistical analysis

Where appropriate, a one-way analysis of variance (ANOVA) was used to compare experimental results with a Dunnett's one-way multiple pairwise comparison test. Depending on the experimental design, a two-way ANOVA was used in conjunction with a Tukey's all pairwise multiple comparison test (SigmaPlot version 12.5; Systat Software, Inc., San Jose, CA).

## Results

### Deleting *luxS* attenuates the mccPDI phenotype

**Figure 1:**
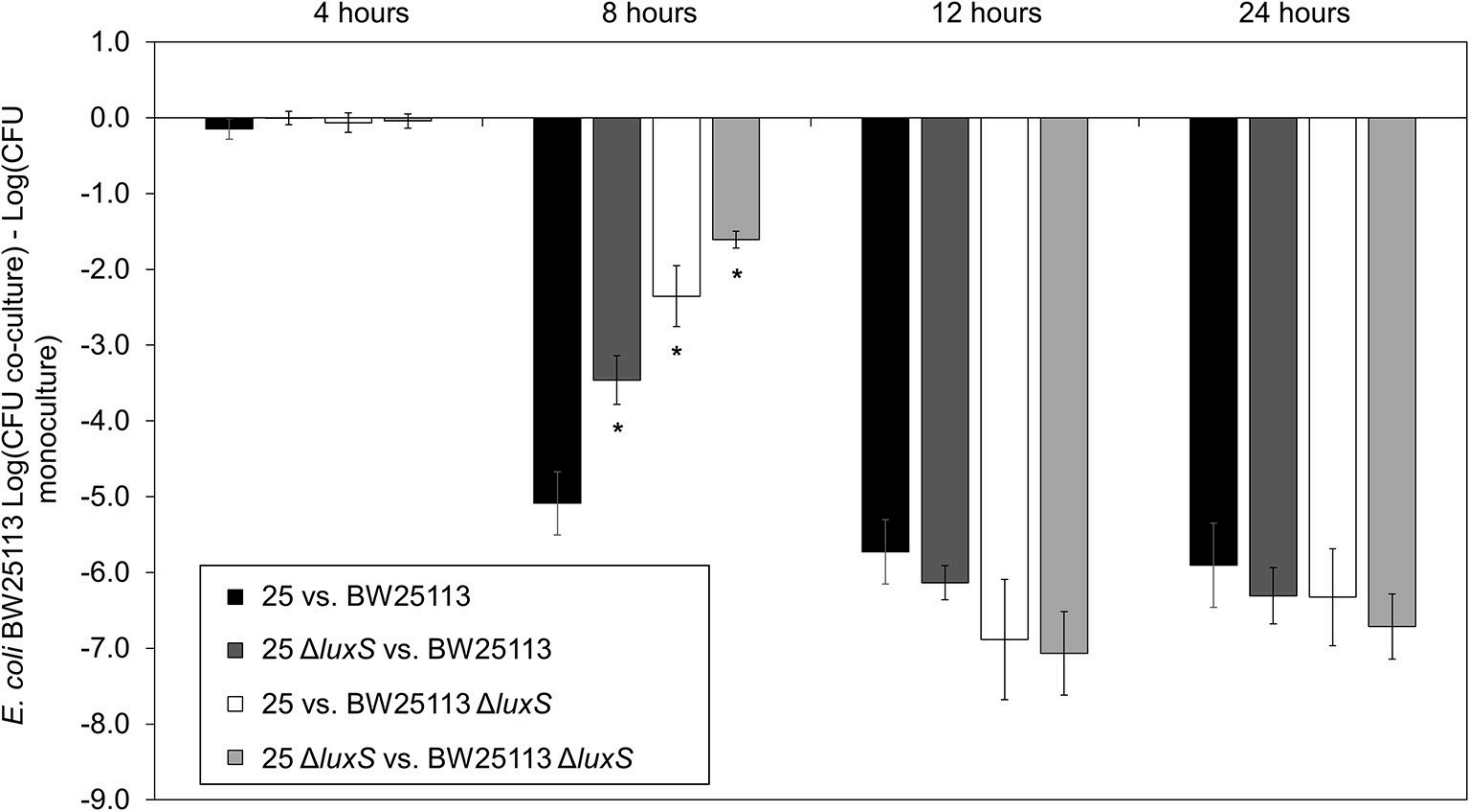
Delayed mccPDI inhibition when *luxS* is deleted. Competition assays between mccPDI-positive *E. coli* strain (25 or 25 Δ*luxS*) and target *E. coli* strain (BW25113 or BW25113 Δ*luxS*) in M9 media for 4, 8, 12, and 24 h. Results are expressed as the difference of mean log CFU during co-culture and mono-culture of the target strain (*n* = 3 independent replicates; error bar = SEM). ^*^P < 0.05 compared to wild-type co-culture (black bars) based on two-way ANOVA.

We conducted co-culture competition assays with *luxS* deletion strains for both the microcin-PDI positive (*E. coli* 25) and susceptible strains (*E. coli* K-12 BW25113; Figure 1). Differences in inhibition were clearly evident for the mid-to-late log growth phase (8 h), which is the same time that there was a 14-fold increase in the abundance of *mcpM* mRNA relative to the 4-h culture of wild-type *E. coli* 25 (Figure S1; Eberhart et al., 2012). Compared to inhibition of BW25113 by the wild-type positive control (at 8 h, Figure 1), eliminating *luxS* from *E. coli* 25 was 1.6-log less effective while eliminating *luxS* from BW25113 reduced the mccPDI phenotype by 2.7-log. When co-culture involved both *luxS* deletion strains, the total reduction in mccPDI phenotype was ~3.5 log relative to the wild-type strain; a finding that was consistent with AI-2 from both strains contributing to a signal for upregulation of mccPDI. After 8 h the effect of *luxS* deletion was no longer observed (Figure 1). As expected, co-culture with the susceptible *E. coli* BW25113 had no negative effects on *E. coli* 25 growth with or without a *luxS* (Figure S2).

**Figure 2:**
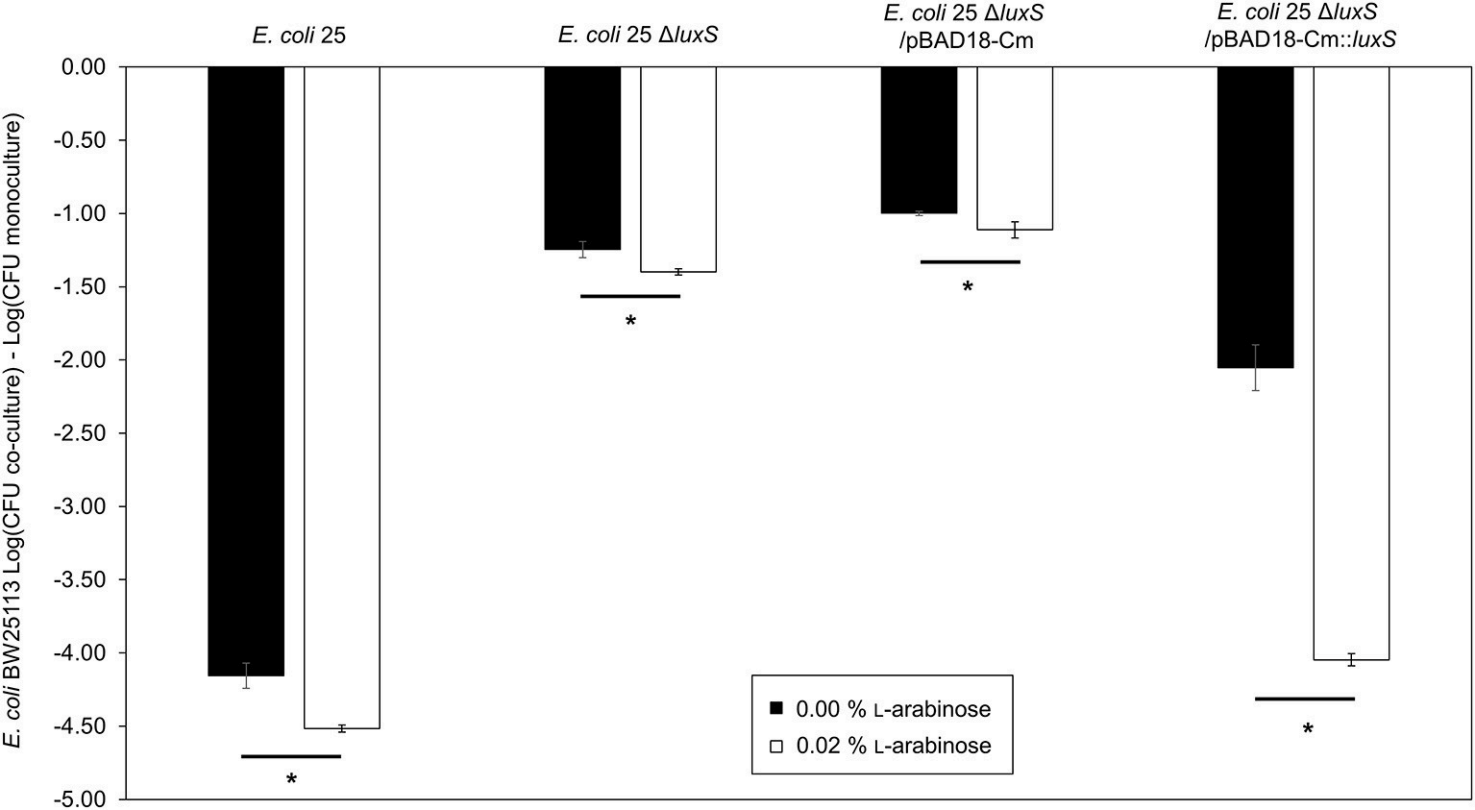
Complementation restores of *luxS* mccPDI phenotype. CFU counts for *E. coli* BW25113 following competition with microcin-PDI producer *E. coli* 25, *E. coli* 25 Δ*luxS, E. coli* 25 Δ*luxS*/pBAD18-Cm, and *E. coli* 25 Δ*luxS*/pBAD18-Cm::*luxS*. Non-induced (black bar) and induced with 0.02% _L_-arabinose (white bar). Results are expressed as the difference in CFU counts of BW25113 grown in co-culture and monoculture (*n* = 3 independent replicates; error bar = SEM). ^*^P < 0.05 compared to wild-type co-culture based on one-way ANOVA.

AI-2 deficient mutant strain *E. coli 25* Δ*luxS* was complemented by using *in trans* expression of *luxS* under the control of an _L_-arabinose inducible promoter, *araBAD* (pBAD18-Cm). A pBAD18-Cm plasmid with no cloned insert was used as a negative control while *E. coli* 25 was used as positive control. Complementation restored the ability of *luxS* deletion strain to inhibit BW25113 compared to the respective un-induced strain (Figure 2, compare first and last bars under *E. coli 25* Δ*luxS*/pBAD18*-*Cm::*luxS*). Adding arabinose to the culture regardless of the presence or absence of the pBAD18-Cm plasmid produced some growth advantage for the *E. coli* 25 strains relative to the susceptible strain (Figure 2, compare the open and filled bars), although this effect did not exceed 0.5 log on average. A western blot confirmed synthesis of the complemented LuxS protein (8 h culture; Figure S3A), and an autoinducer bioassay was consistent with increased production of AI-2 (Figure S3B).

### Deletion of the AI-2 transporter decreases inhibition of mccPDI-susceptible bacteria

**Figure 3:**
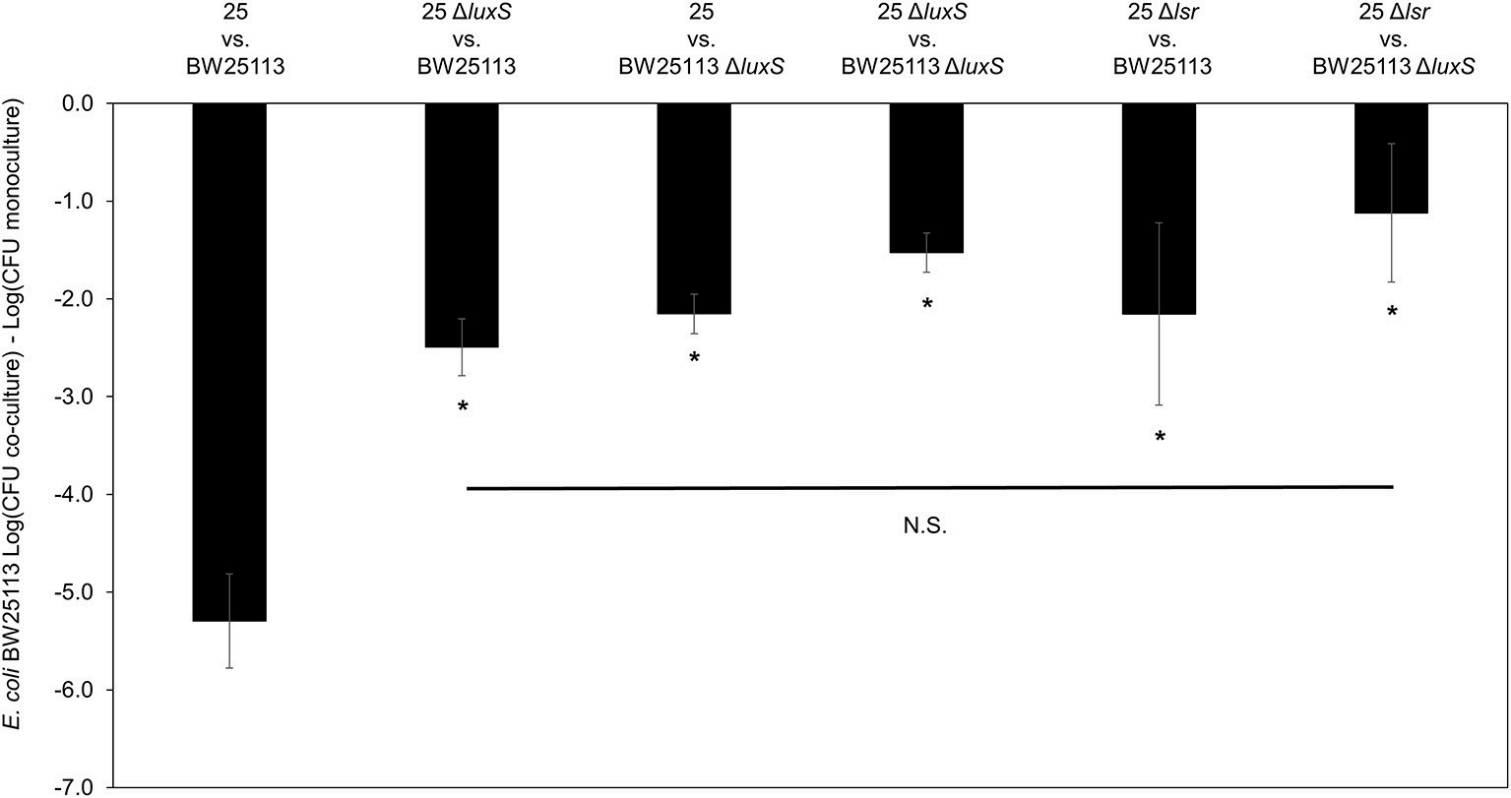
Deletion of AI-2 ABC cassette, *lsrACDBFG* limits the mccPDI phenotype. Competition assay between isogenic mccPDI-producing *E. coli* strains (25, 25 Δ*luxS*, and 25 Δ*lsr*) and target *E. coli* strains (BW25113 or BW25113 Δ*luxS*) in M9 media for 8 h. Results are expressed as the difference of mean log CFU during co-culture and mono-culture (*n* = 3 independent replicates; error bar = SEM). ^*^P < 0.05 compared to wild-type co-culture based on one-way ANOVA.

To further validate the contribution of AI-2 to the regulation of the mccPDI phenotype, we constructed an *E. coli 25* Δ*lsrACDBFG* (*E. coli* Δ*lsr*) mutant (Wang et al., 2005). The *lsr* operon consists of six genes of which *lsrACDB* encodes the ABC transporter, and *lsrF* and *lsrG* are involved in the degradation of AI-2. A separate *lsrR*/*K* operon encodes an uptake repressor and kinase to phosphorylate AI-2, respectively (Li et al., 2007). After 8 h the reduction in inhibition for the Δ*lsr* strain was statistically indistinguishable from the reduction for the Δ*luxS* strain (Figure 3). We further confirmed that deletion of the *lsr* operon or the *lsrR*/*K* operon does not affect production of AI-2 itself (Figure S4).

### RT-qPCR confirms down regulation of *mcpM* in *E. coli 25 ΔluxS*

**Figure 4:**
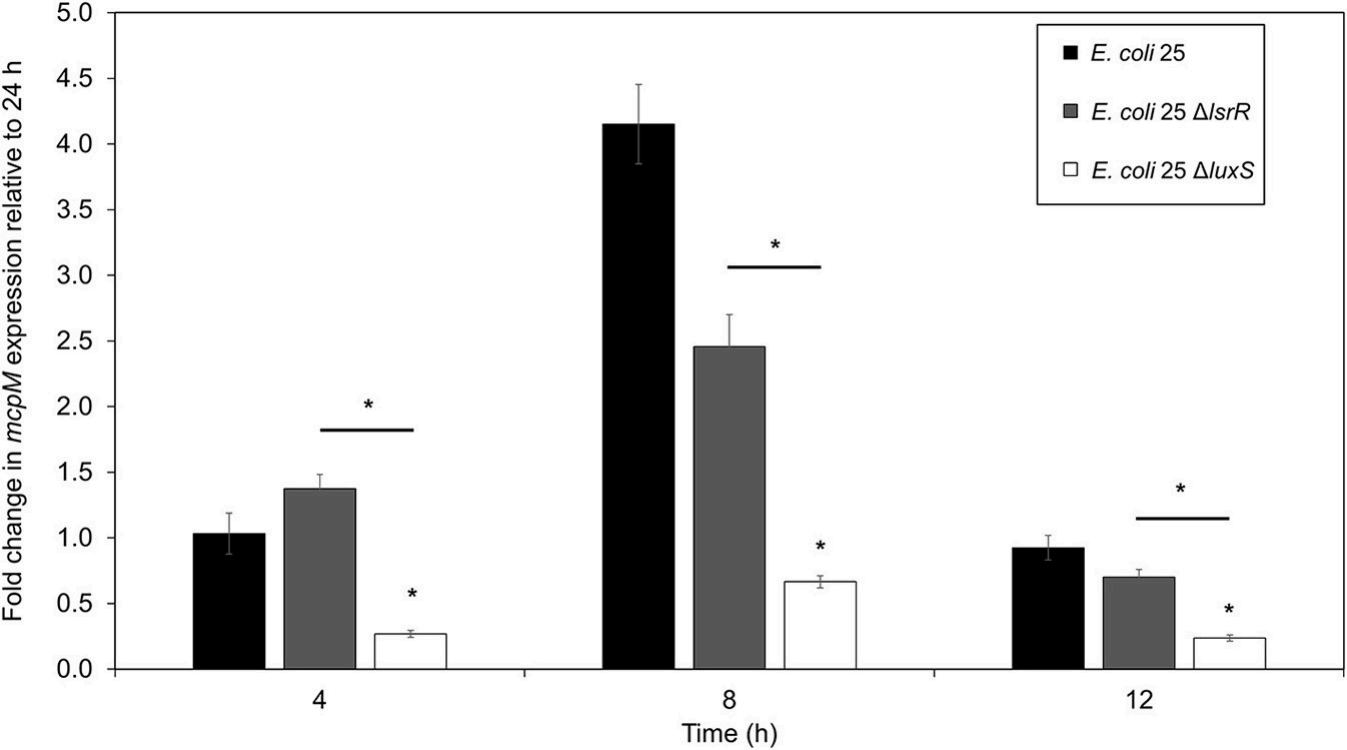
Transcription of *mcpM* is significantly down regulated in AI-2 QS deficient *E. coli* 25 strains. Transcriptional analysis of the mccPDI effector *mcpM* for *E. coli* 25 Δ*luxS, E. coli* 25 Δ*lsrR* mutant and isogenic wild-type strain in M9 media over time by qPCR. Fold change is expressed relative to *mcpM* expression in M9 at 24 h (error bars = SEM; three independent replicates). ^*^P < 0.05 based on two-way ANOVA.

The mRNA for *mcpM* peaks at the mid-to-late log phase growth and declines when cultures enter stationary phase (Figure S1). Under monoculture (1:500 initial dilution) *mcpM* expression differed at 8 h was reduced for Δ*luxS* strains compared to the isogenic wild-type (Figure 4). To verify Δ*luxS* monoculture results, we repeated the experiment from co-culture samples with reduced inoculant (1:1,000 instead of 1:500 to normalize with co-culture experiments) and observed a similar *mcpM* expression pattern, but at later point of 12 h (Figure S5). The pattern of up and down-regulation of *mcpM* expression matches what has been reported previously (Eberhart et al., 2012; Zhao et al., 2017). The AI-2 deficient mutant (Δ*luxS*) clearly exhibits reduction of *mcpM* (Figure 4 and Figure S5) with an overall 5-fold reduction in *mcpM* transcription, consistent with phenotype differences (Figure 1). Furthermore, the strain deficient in AI-2 (Δ*lux*S) has a greater reduction of *mcpM* expression compared to Δ*lsrR* (Figure 4). This suggests that the deletion of the AI-2 uptake regulation gene (Δ*lsrR*) or uptake mechanism Δ*lsr* (Figure 3) can be mitigated through another means of cell entry such as passive diffusion of AI-2 through porins (Galloway et al., 2011).

### *luxS* deletion delays synthesis of recombinant McpM

**Figure 5:**
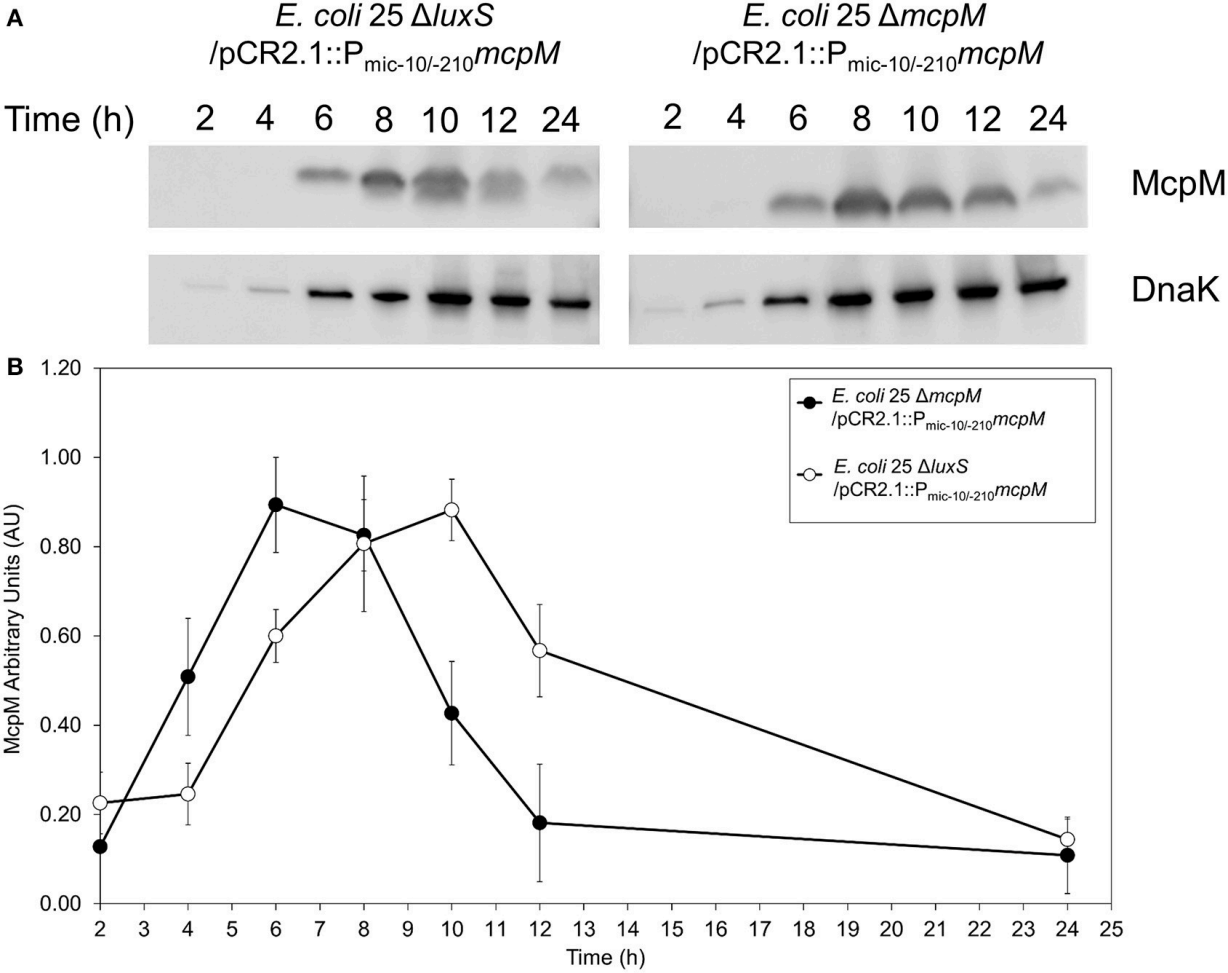
*E. coli* 25 Δ*luxS* mutant causes delay in McpM production. **(A)** Western blot of McpM. Whole-cell lysate samples from *E. coli* 25 Δ*mcpM*/pCR2.1::P_mic−10/−210_*mcpM* and *E. coli* 25 Δ*luxS*/pCR2.1::P_mic−10/−210_*mcpM* complemented strains were collected for every 2 h from 2 to 12 h, and 24 h. Endogenous DnaK served as a loading control. **(B)** Western blot densitometry analysis of McpM. Whole-cell lysate samples from *E. coli* 25 Δ*mcpM*/pCR2.1::P_mic−10/−210_*mcpM* (black circle) and *E. coli* 25 Δ*luxS*/pCR2.1::P_mic−10/−210_*mcpM* (white circle) complemented strains were collected for every 2 h from 2 to 12 h, and 24 h. Endogenous DnaK served as a loading control. Normalization of the McpM against DnaK are represented by arbitrary unit (AU) over 24 h. Error bars = SEM; three independent experiments.

To examine the kinetics of McpM protein synthesis, we used a vector (pCR2.1) with the *mcpM* endogenous promotor (P_mic−10/−210_) coupled with *mcpM* (Zhao et al., 2017). Normalized densitometry of western blot results showed a delay in *E. coli 25* Δ*luxS*/pCR2.1::P_mic−10/−210_*mcpM* recombinant McpM production compared to the strain *E. coli 25* Δ*mcpM*/pCR2.1::P_mic−10/−210_*mcpM* that retained an intact *luxS* (Figure 5). The kinetics of McpM synthesis for both strains mirrored the typical *mcpM* transcription except with a 2-h delay for the Δ*luxS* strain (Figure 5B). The lack of *luxS* does not inhibit the production of McpM because EnvZ/OmpR is still the primary regulator of *mcpM* (Zhao et al., 2017) as confirmed by loss of McpM synthesis with the deletion of the *ompR* (Figure S6A). Deletion of *luxS* also does not affect *ompR* expression, which remains constant through different growth phases (Figure S6B).

### Overexpression of sRNA *micC* and *micF* limits mccPDI

**Figure 6:**
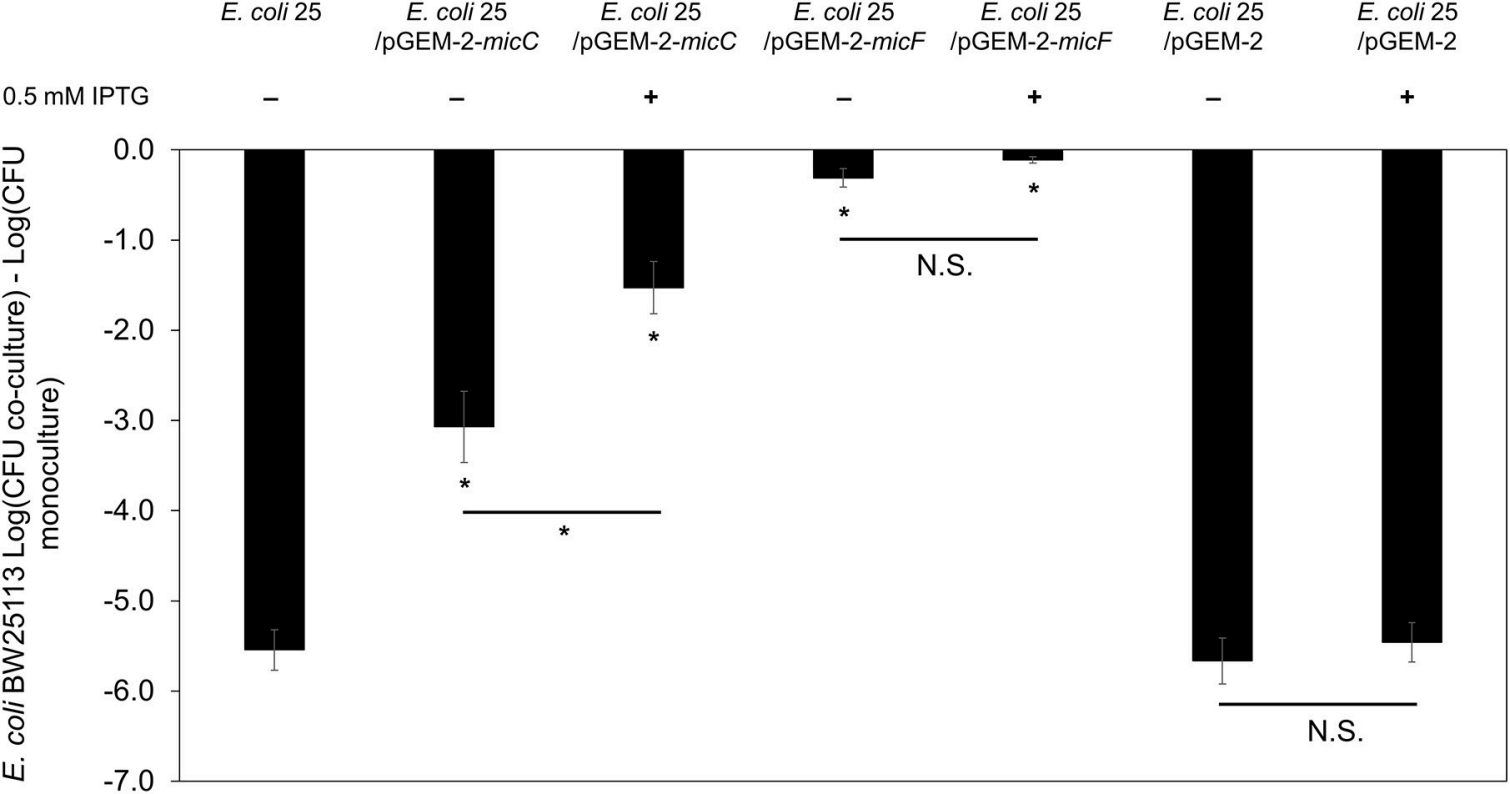
Overexpression of sRNA *micC* and *micF* in PDI-producer strain. The co-culture competition assay of over expression *micC* and *micF* in wild-type *E. coli* 25 (*E. coli* 25/pGEM-2-*micC, E. coli* 25/pGEM-2-*micF, E. coli* 25/pGEM-2) induced with 0.5 mM IPTG against target *E. coli* BW25113 in M9 media for 8 h. Results are expressed as the difference of mean log CFU during co-culture and mono-culture (*n* = 3 independent replicates; error bar = SEM). ^*^P < 0.05 based on one-way ANOVA.

Published work demonstrates that AI-2 QS and LsrR influence the synthesis of the sRNA *micC* (Li et al., 2007), which in turn regulates outer membrane porins (OmpC and OmpF) in a manner similar to the EnvZ/OmpR two-component system (Mizuno et al., 1988). To examine the effects of *micC* and *micF* (another sRNA known to regulate outer membrane porin OmpF in *E. coli;* Delihas and Forst, 2001) in PDI-producer strain, we overexpressed *micC* and *micF* in *E. coli* 25 during competition with strain BW25113. After 8-h co-culture competition it was readily apparent that overexpression of *micC* and *micF* reduced the PDI phenotype significantly (Figure 6). Compared to positive control competition culture (with empty vector pGEM-2; 5-log loss in susceptible BW25113), *micC* overexpression resulted in a 1-log reduction in BW25113 while *micF* overexpression resulted in a complete loss of the PDI phenotype. There was evidence that a “leaky” pGEM-2 vector permitted sufficient *micF* and *micC* expression to reduce the PDI phenotype by 5-log and 2-log, respectively, in the absence of IPTG induction (Figure 6).

## Discussion

The involvement of QS in the regulation of mccPDI was suspected. Eberhart et al. first demonstrated that the expression of the mccPDI effector gene (*mcpM*) increases rapidly during the late-log growth phase and declines rapidly as a culture enters the stationary phase (Eberhart et al., 2012). Zhao et al. demonstrated that without the EnvZ/OmpR two-component regulatory system, *mcpM* expression would not be upregulated (Zhao et al., 2017). The EnvZ/OmpR system functions by sensing the osmolarity of the broth culture (low salt favors upregulation; Zhao et al., 2017). Importantly, even when salt concentration is low the expression of *mcpM* is delayed until late log-growth, after which the expression of *mcpM* is downregulated despite a constant salt concentration (although pH also changes; unpublished results; Eberhart et al., 2012; Zhao et al., 2017). This project concerns the mechanism by which *mcpM* expression is upregulated in the presence of permissible osmotic conditions (low salt) during late log-phase growth.

Microcin production by Gram-negative bacteria is typically triggered by environmental and nutritional factors (Duquesne et al., 2007). Examples include microcin B17, C, E492, and J25 that are regulated by a global regulator (e.g., OmpR and sigma factors), or in response to depletion of nutrient, carbon, and or nitrogen source (de Lorenzo, 1985; Moreno et al., 2002; Socias et al., 2009). Unlike bacteriocins from lactic-acid producing bacteria and for which quorum sensing (QS) is known to play a regulatory role (Drider et al., 2006), to date there have been no reports about the contribution of QS to the regulation of Class I, IIa, or IIb microcin expression. There is some evidence that QS is at least indirectly involved with regulation of other Gram-negative microcins. Piskunova et al. (2017) recently reported that (p)ppGpp can mediate production of microcin C in *E. coli*, presumably as part of the stringent response pathway that is known to interact with quorum sensing (Oh and Cho, 2014). In the case of mccPDI, however, the pattern of upregulation under favorable osmotic conditions reflects what would be expected if regulation was influenced by QS.

### Without QS, upregulation of *mcpM* is compromised

From a broad perspective, QS-regulated bacteriocin production should provide a competitive advantage when resources become limited in the presence of large population of competitors (Blanchard et al., 2016). The PDI-positive strain (*E. coli* 25) used in this study was originally isolated from a cow (Sawant et al., 2011), and by using a neonatal calf model Eberhart et al. showed that the wild-type *E. coli* 25 out competed an isogenic PDI-defective strain (*E. coli* 25 Δ*mcpM* Δ*mcpI*; Eberhart et al., 2014). The “growth phase” of bacteria in the gastrointestinal (GI) tract is likely variable depending on conditions at any given time, but the size of the bacterial population (*E. coli* >10^6^/g feces in cattle) is likely to be within a range that is conducive to QS (Maki and Picard, 1965; Alberghini et al., 2009).

LuxS is necessary for AI-2 synthesis and *E. coli* uses AI-2 for interspecies communication and global gene regulation (Sperandio et al., 2001). *E. coli* can also sense AI-1, AI-3, epinephrine/norepinephrine and other QS molecules (Sperandio et al., 2003; Smith et al., 2004; Walters and Sperandio, 2006; Walters et al., 2006; Connolly et al., 2015; Moreira and Sperandio, 2016) even though it does not produce these signal molecules with exception of AI-3-producing EHEC (Michael et al., 2001; Dyszel et al., 2010; Soares and Ahmer, 2011; Sabag-Daigle et al., 2012). Loss of *mcpM* regulation with deletion of *luxS* (Figure 5) and the combined effect of AI-2 when both *E. coli* 25 and BW25113 are co-cultured (Figure 1) are consistent with AI-2 influencing McpM synthesis. It is presumed that the lower GI tract of a cattle experiences relatively low osmolarity (Brouwer and Van Weerden, 1956) that is conducive to EnvZ/OmpR-mediated upregulation of *mcpM*. In this environment, AI-2 concentration likely provides “fine-tuned” control of expression so that even with permissive osmolarity, McpM is only synthesized when high-density bacterial populations experience conditions conducive to further population growth (e.g., after the host animal ingests a meal).

### Small RNA may play a role in *mcpM* regulation through AI-2 quorum sensing

It is unclear how the concentration of AI-2 regulates *mcpM* expression. We know that decreased AI-2 concentration increases *ompC* expression and represses *ompF* expression during stationary-phase growth (Ren et al., 2004). OmpF is an outer membrane that must be present on susceptible cells before McpM is able to inhibit these cells (Zhao et al., 2015), and as a consequence Zhao et al. speculated that *mcpM* expression should mirror *ompF* expression (Zhao et al., 2017). For *E. coli* LsrR serves as an autoregulatory repressor protein that also regulates *lsrACDB* (AI-2 ATP-binding cassette transporter; Xue et al., 2009). Furthermore, when AI-2 is phosphorylated by LsrK, it subsequently binds to LsrR to regulate other genes associated to biofilm, membrane porins, and sRNA production (Li et al., 2007; Xue et al., 2009). A functional *lsr* AI-2 transport system is AI-2 (*luxS*) dependent (Taga et al., 2001).

Deletion of *lsrR* is associated with the up-regulation of sRNA *micC* through AI-2 signaling (Li et al., 2007). sRNAs *micC* and *micF* bind the mRNA of *ompC* and *ompF* to form MicC-*ompC* and MicF-*ompF* complexes that prevent translation of these mRNAs (Schmidt et al., 1995; Chen et al., 2004; Vogel and Papenfort, 2006). In M9 defined medium, conditions favoring OmpF expression in *E. coli* also favor the synthesis of McpM in *E. coli* 25 at late-log growth phase (Zhao et al., 2017). We speculate that during exponential growth phase, both *micF* and *micC* expression are kept at a base level similar to *ompR* expression (Figure S6B). When the PDI-producer strain reaches stationary growth phase, *micF* and *micC* are up-regulated to reduce synthesis of OmpF and OmpC. We surmise that the sRNA *micF* and *micC* also interact and regulate synthesis of McpM. IntaRNA prediction of pmic_−500/0_*mcpM* (*mcpM*−500 to 0 bp promotor region) sequence against *micC* and *micF* suggests a potential interaction between *mcpM* mRNA (189–241 nt) and *micC* (7–66 nt); *mcpM* (290–345 nt) and *micF* (1–64 nt; Wright et al., 2014). As a result, sRNA *micC* and *micF* could potentially mediate the translation of McpM as suggested in our overexpression experiment (Figure 6), and this would provide a mechanism for down-regulating *mcpM* as the population enters a stationary growth phase.

### Proposed model for McpM regulation

**Figure 7:**
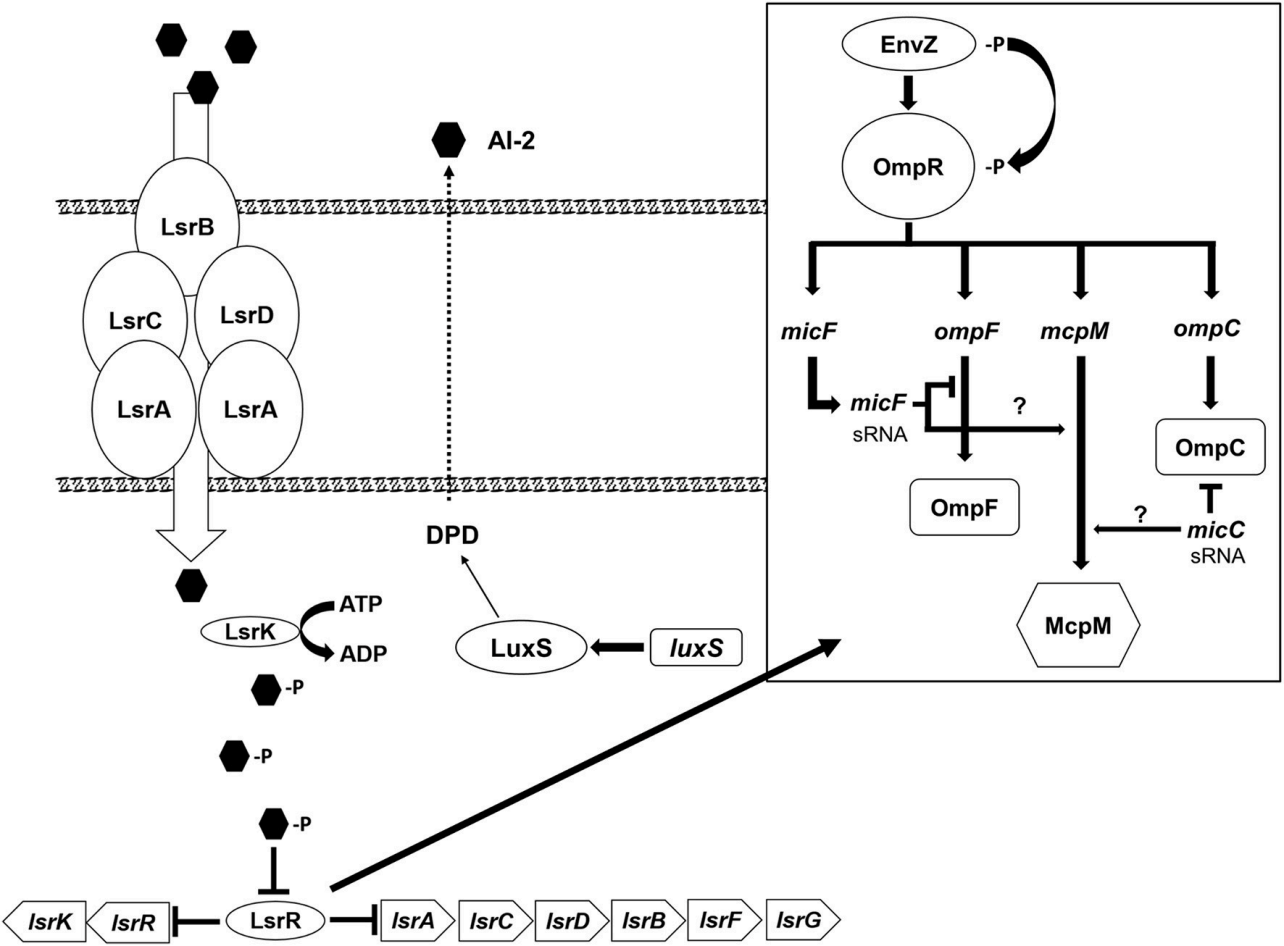
Microcin-PDI regulation model. The proposed regulatory mechanism of *mcpM* through the AI-2 uptake pathway (modified from Li et al., 2007). The AI-2 molecule produced by LuxS is actively transported into the cell by LsrACBD where it is phosphorylated by LsrK. The phosphorylated AI-2 interacts with LsrR and the signal is transduced via LsrR through (1) an unknown mechanism (?) that influences the two component system, EnvZ/OmpR (modified from Delihas and Forst, 2001; Blain et al., 2010) and induces expression of sRNA *micC* and/or *micF* that subsequently bind *mcpM* mRNA to inhibit translation, or (2) via an alternative pathway (?) that regulates transcription of *micF* and/or *micC*.

Disruption of the QS AI-2 synthesis and uptake system in the microcin-PDI producer strain (*E. coli* 25) does not result in complete repression of McpM. Presumably, this is because OmpR interacts directly with the *mcpM* promoter as reported earlier (Figure S6A; Zhao et al., 2017). Herein we propose a McpM regulation mechanism model that incorporates both the EnvZ/OmpR two-component regulatory system and QS AI-2 (Figure 7).

The AI-2 molecule is derived from 4, 5-dihydroxy-2,3-pentadione (DPD), which is catalytically transformed by the LuxS from *S*-ribosylhomocysteine (Schauder et al., 2001). As cellular density increases, AI-2 molecules accumulate in the extracellular milieu. Via the Lsr ABC transporter (comprised of *lsrACDB*), AI-2 in medium is actively transported into permissible cells (Li et al., 2007) although passive diffusion of AI-2 across the cellular membrane is possible (Galloway et al., 2011). The Lsr transporter moves AI-2 into the bacterial cytoplasm where it is phosphorylated by LsrK (Xue et al., 2009). Phospho-AI-2 binds LsrR thereby blocking further repression of *lsr*-transporter genes, which leads to additional AI-2 uptake (Li et al., 2007). At this stage LsrR may bind to a “factor X” that interacts directly with the EnvZ/OmpR two-component system to activate transcription of *micF* [via OmpR which binds to the promoter of *micF* (Coyer et al., 1990; Delihas and Forst, 2001)] and/or directly regulates transcription of *micC* (Chen et al., 2004). sRNA *micC* and/or *micF* in turn block translation of *mcpM* mRNA. Because neither *E. coli* 25 Δ*luxS* nor *E. coli* 25 Δ*lsr* mutants completely or continuously repress the mccPDI phenotype (Figure 1), it is likely that another pathway further contributes to regulation of *micC* and/or *micF* transcription.

## Author contributions

S-YL and DC conceived the experiments. S-YL, ZZ, JA, and JL performed the experiments. S-YL and DC analyzed the results. S-YL and DC wrote the manuscript. All authors reviewed the manuscript.

### Conflict of interest statement

The antibacterial activities of mccPDI are described under US Patent No. 9,492,500 for which DC is an author. The other authors declare that the research was conducted in the absence of any commercial or financial relationships that could be construed as a potential conflict of interest.
