# Supplementary material for: Autoinducer-2 Quorum Sensing Contributes to Regulation of Microcin PDI in Escherichia coli
Source: Front Microbiol. 2017 Dec 22;8:2570. doi: 10.3389/fmicb.2017.02570 (PMC5743794; doi:10.3389/fmicb.2017.02570)
Supplement: Supplementary file 1 [file Table1.pdf]

Supplementary Material

Autoinducer-2 Quorum Sensing Contributes to Regulation of Microcin PDI in *Escherichia coli*

Shao-Yeh Lu, Zhe Zhao, Johannetsy J. Avillan, Jinxin Liu and Douglas R. Call*

***Correspondence:** Douglas R. Call: drcall@wsu.edu

Supplemental Table 1. Primers used in this study

Primer name	Sequence (5'_3')[a, b]	Usage	Reference
**Knockout primers**			
lsrRH1P1	ATGACAATCAACGATTCGGCAATTTCAGAACAGGGAATGTGTGTAGGCTGGAGCTGCTTC	Primer for deletion of *lsrR* gene	Wang et al., 2005
lsrRH2P2	TGATCGGTAACCAGTGCGTTGATATAACCGCCTTTCATTGCATATGAATATCCTCCTTAG		
lsr1P1	CGCTCGGTTTATAAACAGTATTCAGGGGTCAATGTCCTGAGTGTAGGCTGGAGCTGCTTC	Primer for deletion of *lsrACDBFG* operon	Wang et al., 2005
lsr2P2	AGATTCCAGTTTCGCGACACAGGTTTTGTAGTGGGGCGTGCATATGAATATCCTCCTTAG		
lsrK_H1P1	GCGTGAAGGCATTGTTTTATATAATAATGAAGGAACACCGTGTAGGCTGGAGCTGCTTC	Primer for deletion of *lsrK* gene	
lsrK_H2P2	TCCGCCTGCAAAGACTAACGATGAAGGATGAATATTCGACATATGAATATCCTCCTTAG		
lsrR_F	ATGACAATCAACGATTCGGCAATTTCAGAACAGGGAATGT	Primer to check for *lsrR* gene deletion	This study
lsrR_R	TGATCGGTAACCAGTGCGTTGATATAACCGCCTTTCATTG		
lsrK_F	GCGTGAAGGCATTGTTTTATATAATAATGAAGGAACACC	Primer to check for *lsrK* gene deletion	This study
lsrK_R	TCCGCCTGCAAAGACTAACGATGAAGGATGAATATTCGA		
LsrB_FWD	ATGACACTTCATCGCTTTAA	Primer to check for *lsrB* and *lsrH* gene deletion	This study
LsrB_RVS	TCAGAAATCGTATTTGCCG		
SOE_LuxS_F1	CCGCAGGTGGGTAAAGATC	Primer to delete *luxS* gene	This study
SOE_LuxS_R1	TTAGCCACCTCCGGTAATTTT		
SOE_LuxS_F2	TCAGTAAACTATCTTCACAATTAATT		
SOE_LuxS_R2	AGTTTATCCAGGGGTTTGCG		
SacI_luxS_F	ggtggt**GAGCTC**ATGCCGTTGTTAGATAGCTTCA	Primer to complement *luxS* gene with C-terminus 6x His.tag	This study
HindIII_luxS_Rc6His	ggtggt**AAGCTT**CTAATGGTGATGGTGATGATGGATGTGCAGTTCCTGCAACTT		
micC_F	GTTATATGCCTTTATTGTCACAGATTTTAT	Primer to verify *micC* sRNA gene	This study
micC_R	AAAAAGCCCGGACGACTGTT		
micF_F	GCTATCATCATTAACTTTATTTATTACC	Primer to verify *micF* sRNA gene	This study
micF_R	AAAAAAAACCGAATGCGAGGCAT		
pGEM2_F	CTGCAGCCCAAGCTTCC	Primer to conduct inverse PCR of pGEM-2-*micC*	This study
pGEM2_R	GAGCTCGAATTCCGTGTATTC		
K1	CAGTCATAGCCGAATAGCCT	Primer to verify kanamycin resistance gene cassette	Datsenko and Wanner, 2000
K2	CGGTGCCCTGAATGAACTGC		
Kt	CGGCCACAGTCGATGAATCC		
**qPCR primers**			
qPCR_mcpM_FWD	TATGGGACCTCCTTTGATCG	qPCR primer for *mcpM*	This study
qPCR_mcpM_RVS	TCGAAACGCACCAACTCATA		
rpoD_FWD	AGGTATCGCTGGTTTCGTTG	qPCR primer for *rpoD* housekeeping gene	This study
rpoD_RVS	TTCGTACGCAAGAACGTCTG		
qPCR_luxS_FWD	CGTCTACCAGTGTGGCACTT	qPCR primer for *luxS*	This study
qPCR_luxS_RVS	GTGCCAGTTCTTCGTTGCTG		
qompR_FWD	TAGTCAGAGCAACCCGATGC	qPCR primer for *ompR*	This study
qompR_RVS	ACGCGGGTTAAACGGTTTTG	-	-

[a]For gene deletion mutants, the kanamycin primers P1 and P2 (underlined) were added to the 3' end of homologous extension sequences (H1 and H2) of the *E. coli*-specific gene. [b] Restriction enzyme sites are bolded for complementation of genes.

Supplemental Figures

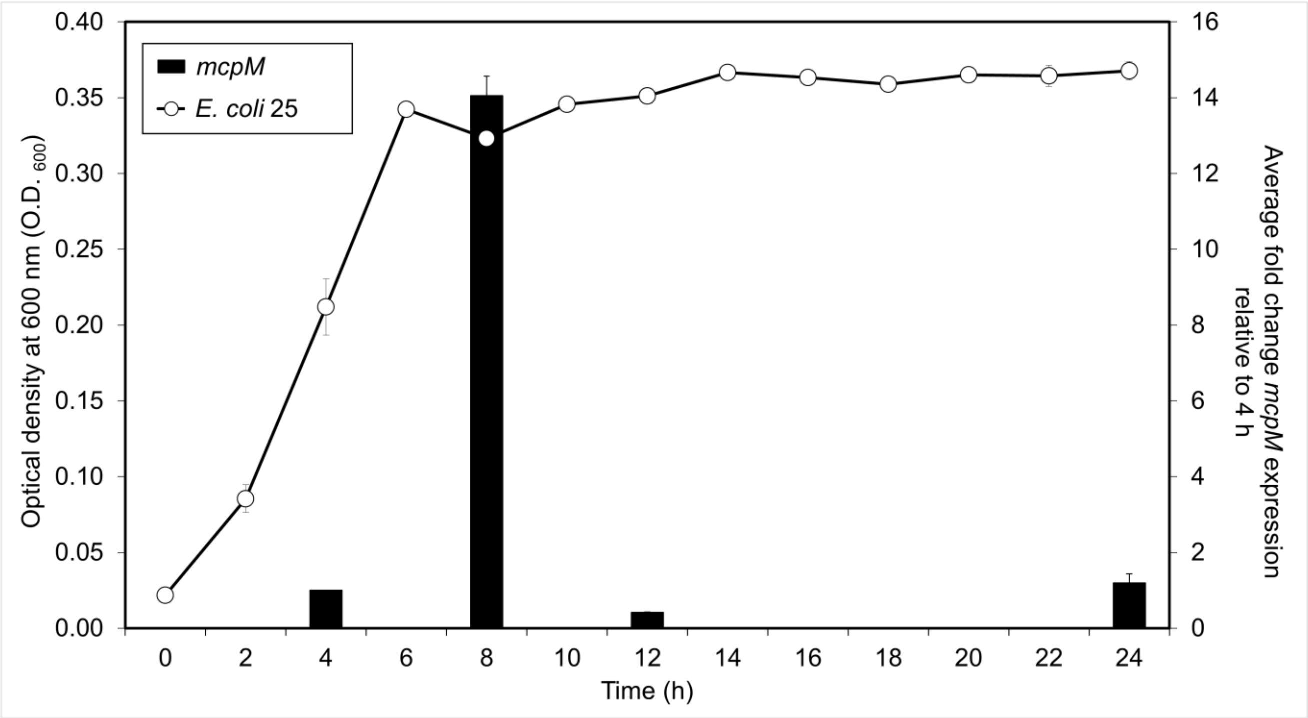

Figure S1. MccPDI up-regulation and down-regulation at mid-to-late exponential growth phase. Wild-type PDI-producer strain growth curve in M9 defined medium for 24 h at 37°C shown in open circle line chart. Black bars indicated average fold change of *mcpM* expression relative to 4 h. PDI- producer strain *E. coli* 25 wild-type grown in M9 defined medium at pH 7 for the 4, 8, 12 and 24 h. The total RNA was extracted to measure relative fold change in mRNA expression of *mcpM*. Error bar = SEM for three biological replicates.

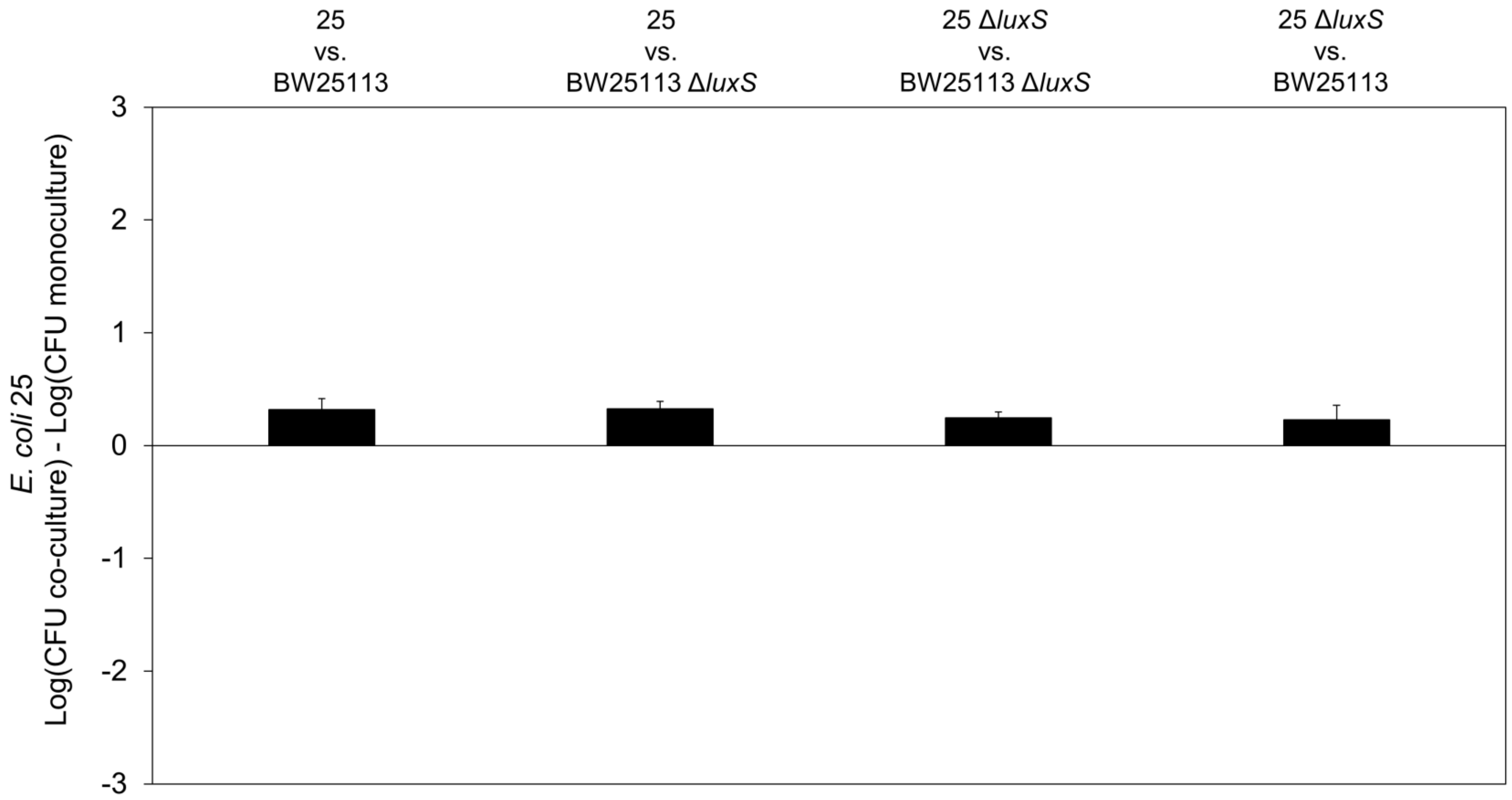

Figure S2. Microcin-PDI producer *E. coli* 25 is not inhibited by *E. coli* BW25113 in co-culture. Competition assay between wild-type and mutant mccPDI-producing *E. coli* strains (25 and 25 Δ*luxS*), and target *E. coli* strains (BW25113 or BW25113 Δ*luxS*) in M9 media for 8 h. Results are expressed as the difference of mean log CFU during co-culture and mono-culture (n=3 independent replicates; error bar = SEM). **P* < 0.05 based on one-way ANOVA.

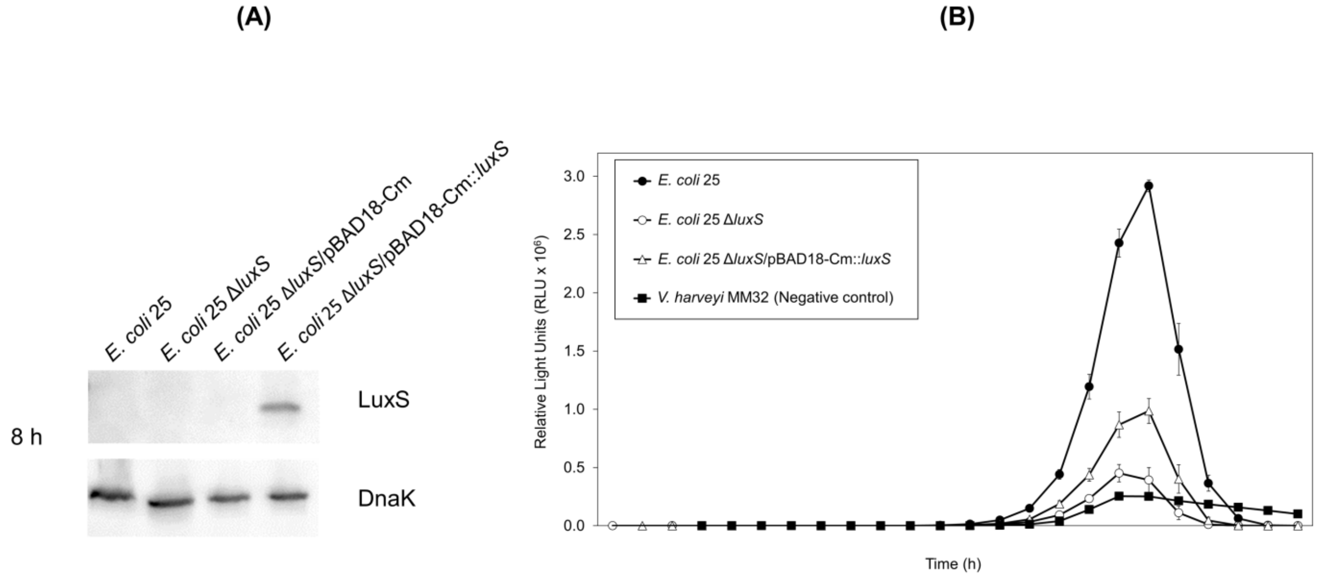

Figure S3. (A) Western blot detection of LuxS. Whole-cell lysate from *E. coli* 25, *E. coli* 25 Δ*luxS*, *E. coli* 25 Δ*luxS*/pBAD18-Cm, and *E. coli* 25 Δ*luxS*/pBAD18-Cm::*luxS* expressing LuxS with c-terminal 6x-His-tag after induction with 0.02% L-arabinose for 8 h. DnaK was used as a loading control. **(B) *E. coli* 25 Δ*luxS* does not produce detectable quantities of AI-2.** Relative light units (RLU) represent the relative concentration of AI-2 in spent media after 24 h as detected by reporter strain *V. harveyi* MM32. Eight hour spent media harvested from *E. coli* 25 (AI-2^+) and QS mutants (*E. coli* 25 Δ*luxS*, AI-2^-; *E. coli* 25 Δ*luxS*/pBAD18-Cm::*luxS*, AI-2^+), and *V. harveyi* MM32 (AI-2^-) were used. Error bars=SEM; 3 independent replicates.

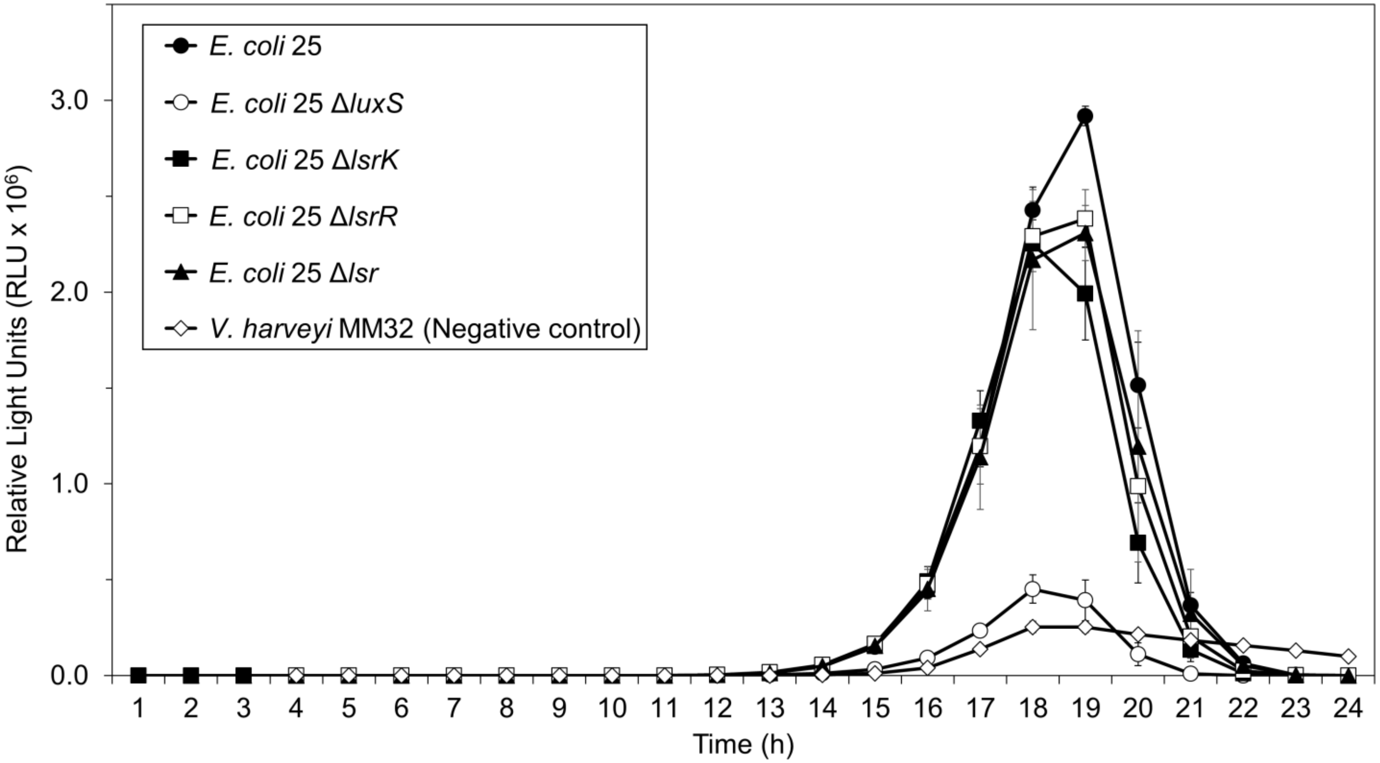

Figure S4. Deletion of *lsr*, *lsrK* or *lsrR* does not affect synthesis of AI-2. Relative light units (RLU) indicate the relative concentration of AI-2 in supernatant samples (8 h). Samples were added to reporter *V. harveyi* MM32 and incubated 24 h before collecting luminescence data. This result shows comparisons for *E. coli* 25 wild-type (AI-2^{+}) (positive-control), *E. coli* 25 Δ*luxS* (negative control) and other quorum-sensing mutants (*E. coli* 25 Δ*lsrK*, *E. coli* 25 Δ*lsrR*, *E. coli* 25 Δ*lsr*). Error bars = SEM; 3 independent replicates).

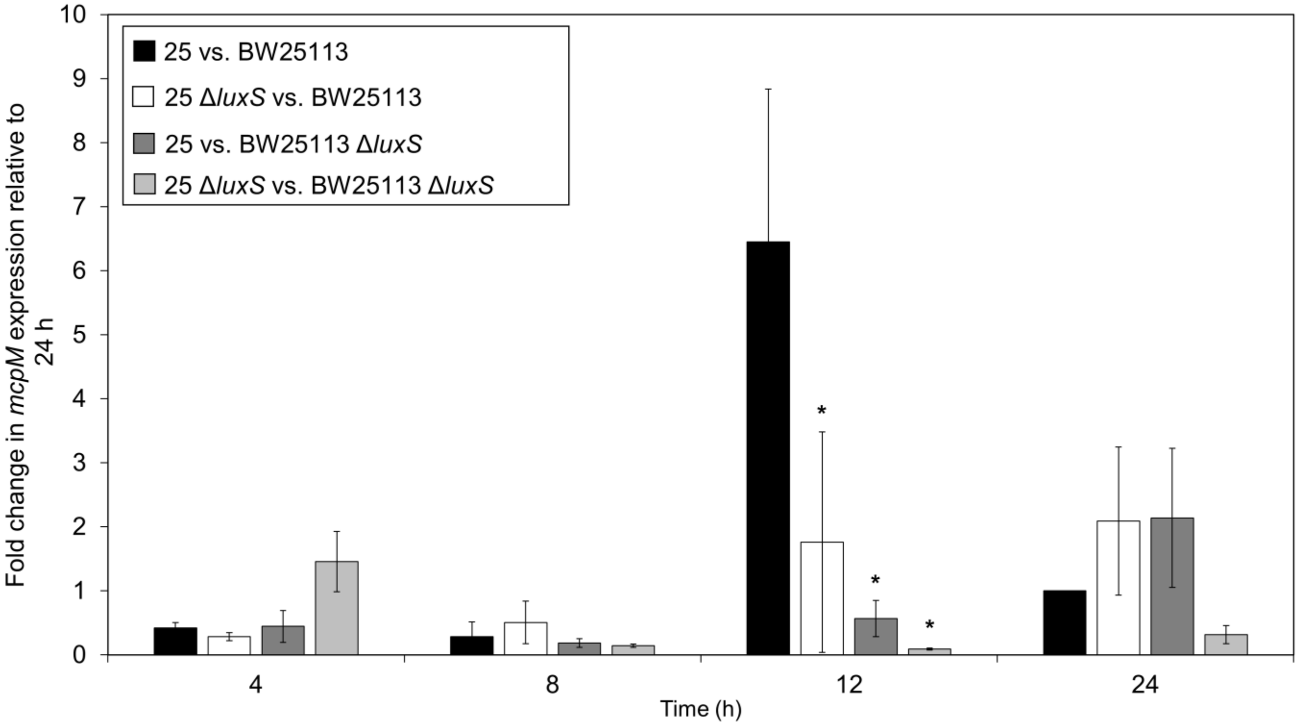

Figure S5. The microcin-PDI phenotype is linked to quorum sensing AI-2. Transcriptional analysis of *mcpM* for *E. coli* 25 wild-type and isogenic Δ*luxS* mutant co-cultured with BW25113 or BW25113 Δ*luxS* in M9 media by qPCR. The comparisons between wild-type co-culture (black), a single strain AI-2 deficient co-culture (white, *E. coli* 25 vs *E. coli* BW25113 Δ*luxS*; dark grey, *E. coli* 25 Δ*luxS* vs. *E. coli* BW25113), and all AI-2 negative (light grey) are represented in a bar graph over time point 4, 8, 12 and 24 h. Fold change is expressed relative to *mcpM* expression in M9 at 24 h (error bars = SEM; 2 independent replicates). *$P < 0.05$ based on two-way ANOVA.

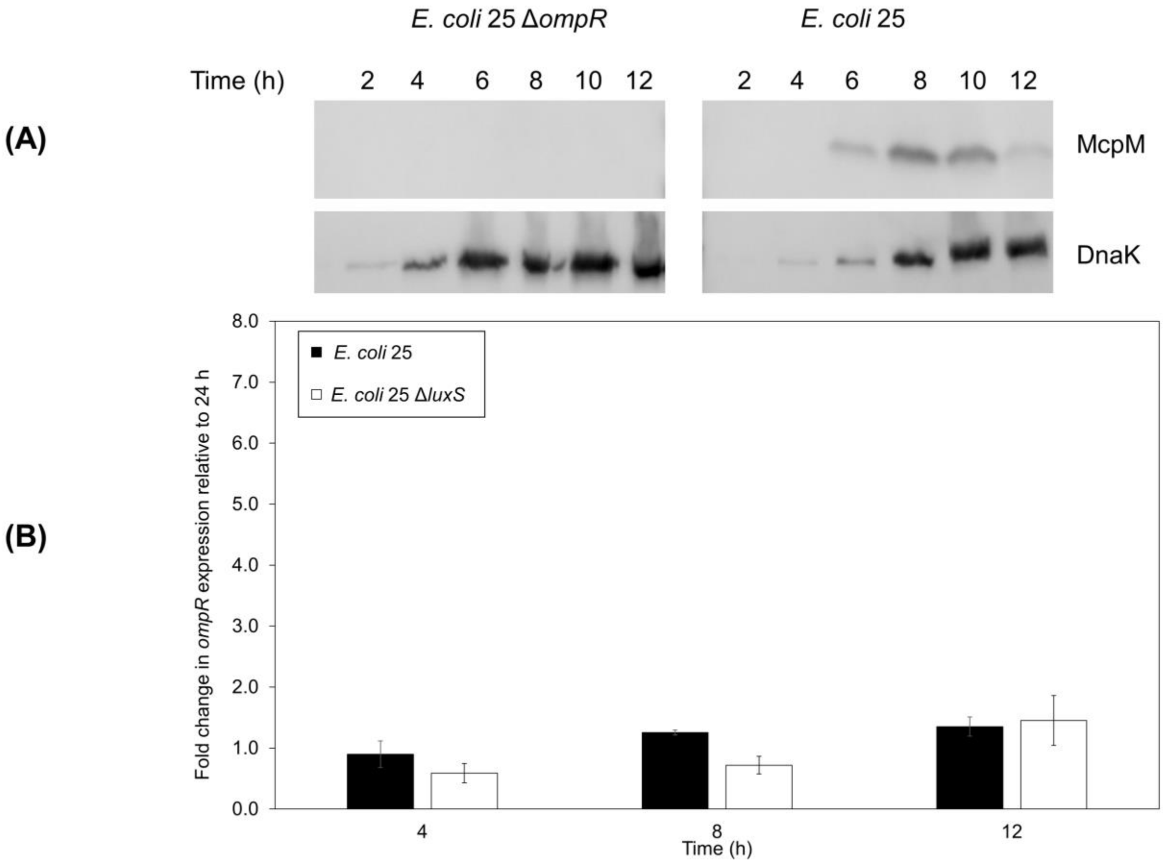

Figure S6. (A) Whole-cell lysate samples from *E. coli* 25 Δ*ompR* and *E. coli* 25 wild-type. Samples collected for every 2 h from 2 – 12 h. Endogenous DnaK served as a loading control. **(B)** Transcriptional analysis of *ompR* for *E. coli* 25 and isogenic Δ*luxS* mutant in M9 over the time course of 4, 8, 12, and 24 h by qPCR. Fold change is expressed relative to *ompR* expression in M9 at 24 h (error bars = SEM; 3 independent replicates). *$P < 0.05$ based on two-way ANOVA.
